# Synthetic Biology of Natural Products Engineering: Recent Advances Across the Discover–Design–Build–Test–Learn Cycle

**DOI:** 10.1021/acssynbio.4c00391

**Published:** 2024-08-20

**Authors:** Jonathan Foldi, Jack A. Connolly, Eriko Takano, Rainer Breitling

**Affiliations:** Manchester Institute of Biotechnology, Department of Chemistry, School of Natural Sciences, Faculty of Science and Engineering, University of Manchester, Manchester M1 7DN, United Kingdom

**Keywords:** natural products, biosynthetic gene clusters, synthetic biology, genome mining, strain engineering, machine learning

## Abstract

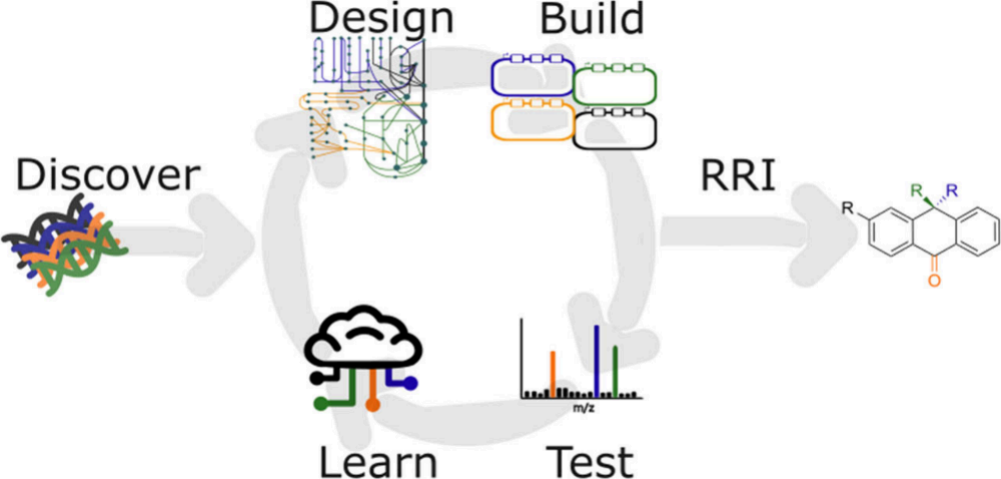

Advances in genome engineering and associated technologies have reinvigorated natural products research. Here we highlight the latest developments in the field across the discover–design–build–test–learn cycle of bioengineering, from recent progress in computational tools for AI-supported genome mining, enzyme and pathway engineering, and compound identification to novel host systems and new techniques for improving production levels, and place these trends in the context of responsible research and innovation, emphasizing the importance of anticipatory analysis at the early stages of process development.

## Introduction

**Figure 1:**
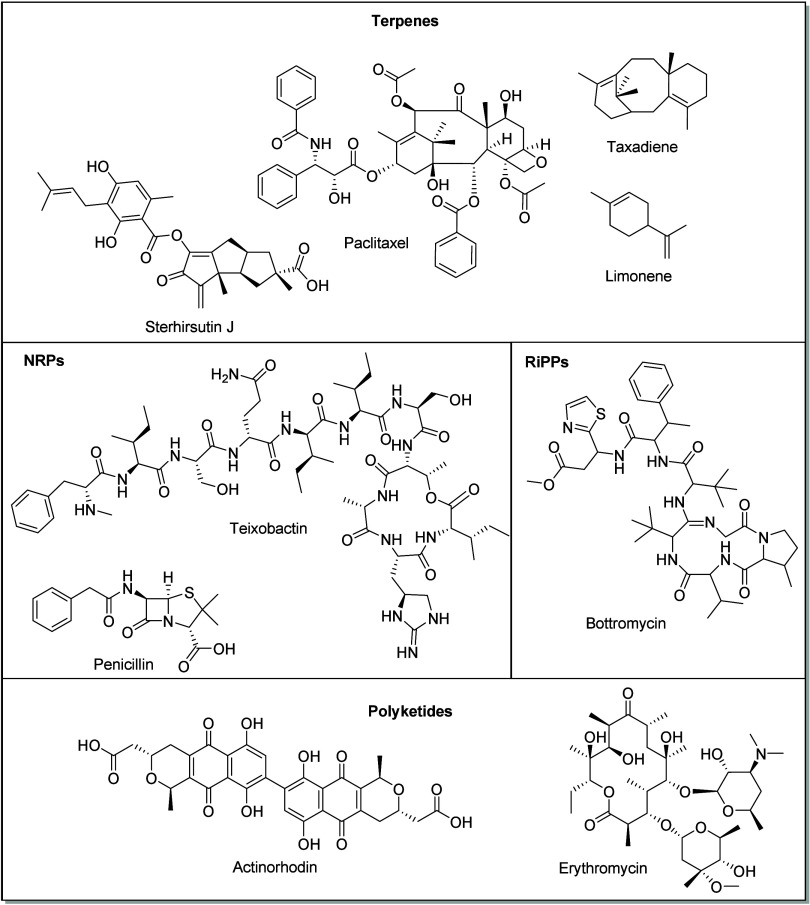
Example compounds from four of the major classes of NPs: terpenes, nonribosomal peptides (NRPs), ribosomally synthesized and post-translationally modified peptides (RiPPs), and polyketides.

Natural products (NPs), also known as secondary metabolites, form the basis for many products of biotechnology, including antimicrobials, chemotherapeutic agents, and pesticides.^1^ This has been the case throughout human history, with the use of natural remedies being documented as far back as the Mesopotamians in 2600 BCE.^2,3^ These natural sources are still just as relevant, as from 1981 to 2019, 64% of antimicrobials (excluding vaccines) either were NP-derived, contained an NP pharmacore, or were synthetic NP mimics.^4^ Since the “Golden Age” of antibiotic discovery in the mid-20th century, the discovery of new antibiotics has dropped off dramatically.^5^ The need for novel antimicrobials remains paramount, as antimicrobial resistance is one of the major global health challenges for this century, already causing an estimated 4.95 million deaths globally in 2019 and predicted to increase substantially in the future.^7,8^ The varied structures of NPs, along with the high levels of stereochemistry relative to synthetic compounds, are highlighted in Figure 1.

In order to address this issue, significant research was directed at NP discovery and engineering after advances in genomics had reopened the field as a viable alternative to high-throughput screens of combinatorial chemical libraries.^9^ Underlying much of this genomic revolution is the fact that the genes which encode the biosynthetic machinery for the production of a natural product are typically colocated in close proximity in the genome of a bacterium or fungus, forming biosynthetic gene clusters (BGCs) that serve as functional evolutionary units for the horizontal transfer of biosynthetic capabilities. This has allowed a relatively straightforward linking of natural products to genomic data that has made it easier to avoid the duplication issues which frequently arose from screening-based discovery methods, thus streamlining the discovery process.^10^ These efforts have led to high numbers of unique natural products being characterized, with four widely used and steadily growing NP repositories from across academia and industry that contain only deduplicated data (Table 1). It is worth highlighting the Collection of Open Natural Products (COCONUT), which was created specifically to address the overproliferation of NP databases, many of which are insufficiently maintained after their initial publication.^11^

**Table 1:** Repositories for Natural Products

name	number of NP entries	notes	ref
Natural Products Atlas	32,552 NPs	only microbial NPs	(13)
SuperNatural III	449,058 NPs and derivatives	includes NPs and derivatives of NPs	(14)
Dictionary of Natural Products	>340,000 NPs	only commercially available	
COCONUT	>400,000 NPs	compilation of 54 open-access NP databases	(15)

However, a large fraction of the biosynthetic potential remains underexplored in its biochemical detail: when analyzed at the level of gene cluster families (GCFs), collections of BGCs sharing a similar architecture and expected to produce similar NPs, it is estimated that fewer than 3% of GCFs have had their biosynthesis routes experimentally characterized and documented in a standardized way.^16^ This can be due to a variety of challenges, the most important being the limited scalability of the experimental elucidation of biosynthetic pathways; however, even a well-established NP like paclitaxel, which is used as a chemotherapeutic agent for thousands of patients, had its biosynthetic pathway elucidated in its entirety only very recently due to its biosynthesis genes not being clustered, as can often be the case for such plant-derived NPs.^17^

**Figure 2:**
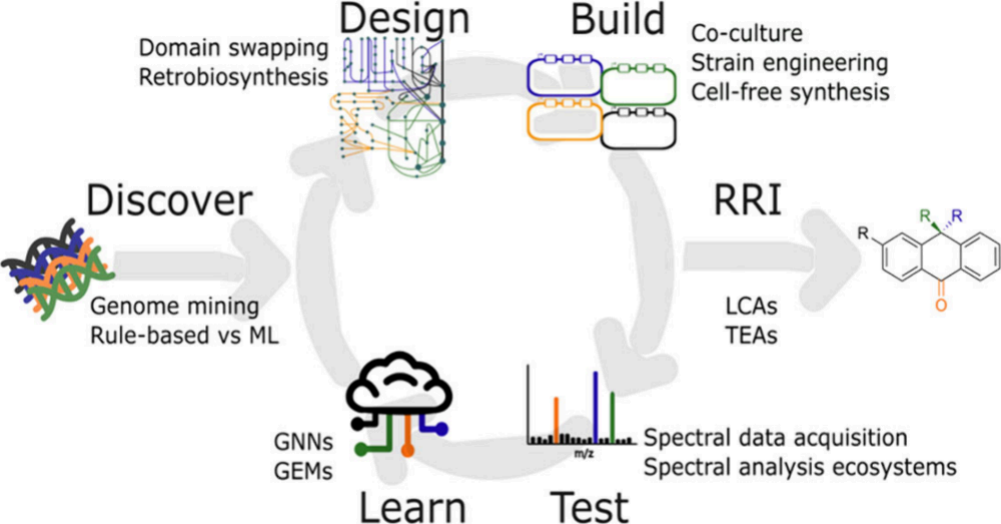
Diagram showing the Discover–Design–Build–Test–Learn cycle for NP engineering, with a few key aspects of each stage listed. GNN, graph neural network; GEM, genome-scale metabolic model; LCA, life cycle analysis; TEA, techno-economic assessment.

A typical route to NP discovery is the following: a BGC of interest, identified through genome mining, is endogenously or heterologously expressed *in vivo*, and its associated NP is purified and characterized through spectral analysis.^18,19^ Our review reframes this standard workflow into an adapted Discover–Design–Build–Test–Learn cycle to highlight the existing and future opportunities for synthetic biology in this field. Additionally, we review recent progress in the development of responsible research and innovation practices that are commonly applied in synthetic biology research and could make a valuable contribution in natural product development (Figure 2).

## Discover

A necessary first step in most NP engineering workflows is the use of genome mining to discover the genes responsible for the biosynthesis of the NPs. In microorganisms, most NPs are coded for by BGCs which can be highly variable in the number of genes they contain.^20^ The number of BGCs present in a genome can also vary, with some microorganisms having over 80 BGCs in their genomes.^21^ Recent advances in long-read sequencing and culturing have offered means to navigate the estimated 99% of microbial species which are not culturable under standard laboratory conditions.^22^ The novel antibiotic teixobactin was discovered in this manner using iChip technology to culture *Eleftheria terrae*, which previously had not been culturable.^23^ Even though this drug has yet to live up to the initial high expectations, the iChip technology continues to be used for discovering novel BGCs.^24^ This technique utilizes a small device with hundreds of pores which sequester microorganisms to grow individually while exchanging nutrients with their natural environment *via* diffusion through a semipermeable membrane before being brought back to the lab for testing. Environments which harbor these unculturable microorganisms, such as deserts and wastewater treatment plants, have recently been shown to harbor a wide swath of microorganisms which can now be sequenced and exploited.^25,26^

To discover the BGCs hidden in these genomes, a suite of tools can be used. AntiSMASH,^27^ the most widely used BGC discovery tool, can annotate sequences from a variety of kingdoms and output a list of BGCs and the corresponding NP classes to which their expected products belong. Different flavors of antiSMASH account for differences in BGCs across kingdoms. For example, plantiSMASH accounts for several distinct aspects of plant BGCs relative to microbial BGCs (unique enzyme families, unclustered enzymatic pathways, higher variability in intergenic distances) to enable more sensitive detection.^28^ The antiSMASH ecosystem also includes tools for interrogating samples from different biomes, such as rhizoSMASH and gutSMASH, which enable BGC discovery in the rhizosphere and gut microbiome, respectively. These tools allow for the detection of BGCs across a wide variety of samples and potential use cases, highlighting the varied potential of natural products across several different sectors. One limitation, however, is that antiSMASH is a rule-based tool and thus does not perform as well as ML-based tools in discovering novel BGCs and unclustered pathways, in contrast, *e.g.*, with GECCO, which found almost twice as many BGCs as antiSMASH in the proGenomes2 database, though a direct comparison has not yet been done using the newest release of antiSMASH.^29^ Similarly, SanntiS, another ML-based tool, can detect more novel BGCs than antiSMASH and was shown to have the highest precision–recall performance of these three genome mining tools on a frequently used “9 genomes” dataset.^30^ Phylogenetic analysis of BGCs can be performed with tools such as BiG-SLICE and CORASON, which can create GCFs to enable comparison across clusters. These pieces of software are listed in Table 2, along with frequently used repositories of annotated BGCs.

**Table 2:** Software Tools for Genome Mining and Analysis and Repositories of Annotated BGCs

name	type of tool	organisms searched	notes	ref
antiSMASH	genome mining	bacteria, fungus, archaea, plant	flagship genome mining tool for NPs; different flavors depending on the organism/biome of choice	(27)
PRISM 4	genome mining	bacteria	emphasis on predicting chemical structures of predicted BGC products	(31)
EvoMining	genome mining	bacteria	focus on overlooked enzyme classes through an evolutionary lens	(32)
GECCO	genome mining	bacteria	uses a specific subset of ML, conditional random field, which makes it more interpretable than neural network “black box” tools	(29)
SanntiS	genome mining	bacteria	uses an artificial neural network and aims to better identify less-characterized BGCs	(30)
BiG-SLiCE	phylogenetic analysis	bacteria, archaea	generates gene cluster families from BGCs	(33)
CORASON	phylogenetic analysis	bacteria	shows evolutionary relationships between BGCs within a gene cluster family using a multilocus phylogeny	(34)
MIBiG	repository	bacteria, archaea, fungi, plants	frequently updated and validated by subject matter experts	(35)
antiSMASH-DB	repository	bacteria, archaea, fungi	not as well curated as MIBiG; depends on the quality of the antiSMASH analysis of a genome	(36)

## Design

Understanding the biosynthetic pathway of an NP is critical to designing efficient biosynthesis strategies, in addition to engineering changes to the product. The modular nature of many NP biosynthesis pathways allows for domain swapping as a popular method of engineering.^37,38^ This approach can help in understanding the functions of specific enzymes in biosynthesis^39^ or to create novel compounds by mixing enzymes from different biosynthesis pathways in order to generate novel NP derivatives.^40,41^ This strategy was recently used in a high-throughput screen for the synthesis of the nonribosomal peptide pyoverdine, in which over 1000 unique domains were substituted into the biosynthetic pathway, resulting in the identification of 16 unique NPs.^42^ An alternative approach using domain swapping created a fluorescent biosensor reporting on solubility of swapped modules to find functional recombinant polyketide synthases (PKSs).^43^ This approach offered an exciting new means of a high-throughput screen for soluble PKS variants, although it should be noted that solubility alone does not mean that such fusions will be functional.

These strategies make use of the natural biosynthesis pathways for NPs, but when a desired compound cannot be produced by an existing natural pathway, tools for retrobiosynthesis can be used to design a pathway. Retrobiosynthesis is a key tool in pathway engineering which takes a chemical compound of interest and breaks it into smaller constituent parts in order to build a bottom-up metabolic pathway to produce the end product using metabolites of the chassis strain.^44,45^ BioNavi-NP is a retrobiosynthesis software tool that focuses on NP pathway prediction. Compared with general metabolic engineering tools such as RetroPathRL, BioNavi-NP had a 13% higher pathway hit rate accuracy on the LASER test dataset and was performed in almost an order of magnitude less time.^46^ It is able to accurately predict the biosynthesis pathways for diverse NPs, including large structures such as sterhirsutin J.

For the selection of individual enzymes to catalyze the reactions within a pathway, tools such as Selenzyme can be used, which returns a ranked list of candidate enzymes, informed by parameters such as their phylogenetic distance from the host organism.^47^ This approach provides a key benefit of being able to propose candidate enzymes for reactions for which no matching enzyme is known yet. Optimization of the proposed enzymes for the target reaction can utilize the latest insights from protein folding algorithms, with AlphaFold serving as a prime example.^48^ The protein-modeling approach from these algorithms has opened up the ability to rationally design mutants based on the proposed structures of proteins in biosynthesis pathways which have not previously been experimentally determined.^49^

## Build

A continuing challenge in the field of NPs is that the majority of BGCs are not endogenously expressed in standard laboratory conditions, limiting efforts to characterize the NPs they synthesize.^50,51^ This has led to heterologous expression being a widely used technique to investigate BGCs and their corresponding NPs. This has especially been the case with the *Streptomyces* genus, which is the most frequently studied bacterial genus for NPs due to the high number of BGCs it encodes.^52,53^ Specific strains of *Streptomyces*, such as *Streptomyces albus* due to its small genome, are frequently used as heterologous hosts for BGCs from environmental *Streptomyces* strains which are not cultivatable in the laboratory.^54^ This work has been aided by advances in CRISPR-Cas9 systems, which allow for rapid genetic engineering of these strains, and has recently has expanded to multiplexed base editing systems as well.^55^ In a promising recent study, the authors used coevolutionary analysis to identify genes coevolving with polyketide NP production and found a gene cluster encoding for the cofactor pyrroloquinoline quinone (PQQ), which is strongly linked to polyketide synthesis.^56^ Overexpression of the *pqq* operon could be introduced to 11 *Streptomyces* species and caused an increase in the production of numerous known NPs and, more importantly, a large number of previously uncharacterized NPs.

Advances in gene editing have also allowed the exploitation of cross-kingdom heterologous hosts, such as yeast strains being used for expression of plant BGCs due to the relative similarity of their subcellular compartmentations.^57,58^ The model yeast *Saccharomyces cerevisiae* has often been used for the heterologous expression of plant BGCs, with several examples of medicinal NPs being synthesized.^59,60^ Nonmodel yeasts can also be leveraged as heterologous hosts; for instance, *Yarrowia lipolytica* is popular because of its high capacity for lipid storage, though as with all engineered hosts there is a significant metabolic burden from accumulation of target compounds.^61^ Genetic engineering advances promise to alleviate some of these issues, as a multiplex base-editing system was recently shown to have higher editing efficiency than wild-type CRISPR-Cas systems in *Y. lipolytica* and thus promises increased control in strain engineering to optimize expression.^62^ These genetic engineering strategies increase the breadth of compounds the yeast can produce heterologously, a list that already includes important commercial terpenoids such as limonene and taxadiene.^63^

Genetic engineering is not the only mechanism for activating silent BGCs. Modifying specific environmental triggers including temperature,^64^ pH,^65^ and quorum sensing^66^ has been shown to induce synthesis and allow for the characterization of novel products. The cellular environment can also be manipulated biologically by using coculture techniques to induce expression.^67^ In this experimental design, which mimics the natural environmental conditions of microbial interactions, the two participating species are frequently categorized as inducer and producer strains, with the former secreting a molecule which instigates the production of an NP by the latter.^68^ A surge of new insights into these interactions has been provided by recent advances in the study of microbiomes, and similar techniques of coculturing and genetic engineering can be applied to further NP engineering efforts.^69−71^ Coculturing also can take advantage of the compartmentalization of pathway steps in different microorganisms, thus taking advantage of different strains’ metabolic capacities^72,73^ (Table 3). When needed, this interstrain compartmentalization can be forced, *e.g.*, by restricting the producer strain within hydrogel beds through which the inducer’s products can diffuse.^74^ Recent modeling work has shown that it should be possible to stably maintain highly burdensome biosynthetic pathways distributed across the members of a community through “dynamic division of labor” by horizontal gene transfer.^75^

**Table 3:** Recent Examples Highlighting Different Build Strategies

build strategy	product	conditions	ref
coculturing	phenylpropene	tripartite coculture of *E. coli* to spread metabolic burden across organisms	(76)
coculturing	(*S*)-norcoclaurine	*Scheffersomyces stipitis* produces shikimate, which *S. cerevisiae* converts to (*S*)-norcoclaurine	(72)
strain engineering	armeniaspirols	knockout of competing polyketide cluster in *Streptomyces* sp. A793 and introduction of heterologous fatty acid synthase to ease bottleneck of precursors from primary metabolism	(77)
strain engineering	citramalate	transposon-guided copy number engineering of *Issatchenkia orientalis*, which can grow at low pH	(78)
strain engineering	pamamycin	pamamycin-resistant *Streptomyces albus* J1074 underwent transcriptional engineering by screening of a promoter library	(79)
cell-free synthesis	salivaricin B	one-pot reaction of engineered *E. coli* extract with chaperone proteins, precursor peptides, and tailoring enzymes	(80)

Though not yet viable on industrial production scales, cell-free systems offer an intriguing means to circumvent issues inherent with cellular expression, such as diversion of energy toward cellular maintenance and unknown genetic regulation networks.^81,82^ A key benefit for natural product engineering which cell-fee systems provide is the ability to utilize certain cofactors and byproducts whose expression or accumulation can be toxic to the cell, such as the analogs of *S*-adenosylmethionine used to synthesize caffeine.^83^ Large and complex synthesis proteins, such as nonribosomal peptide synthetases, can also be too metabolically burdensome for some cells but can be used in cell-free systems.^81,84^ Cell-free systems can also enable quicker benchtop production of certain NPs, such as thiopeptide scaffolds.^85^ One example that highlights this speed utilized the cellular extract of an engineered strain of *E. coli* which lacked several peptidases and protease genes, whose products had inhibited the cell-free synthesis of the target peptide.^80^ This approach, termed unified biocatalysis, relied on a mix of precursor peptides, relevant tailoring enzymes, bespoke chaperone proteins, and cellular extract in a one-pot reaction. It yielded an increase in the target RiPP titers in a few hours, which also enabled researchers to produce several derivatives of the target compound. Despite this promise, it is important to acknowledge that a lack of thorough documentation of the components in some of these systems leads to the field remaining inscrutable at times.^86,87^

## Test

After an expression system for an NP has been built, its expression must be tested by characterizing the produced chemical compound. The field of NP research relies heavily on chemical analysis in order to characterize and quantify the production of the desired compounds. Tandem mass spectrometry (MS^2^) is frequently used for this purpose, to isolate and identify chemical compounds. However, this method is often challenged by the fact that many of the most interesting products are being characterized for the first time at the time of discovery, and thus, authentic standards—by definition—are not yet available. Even for previously characterized NPs, the analysis in a new producer can be complicated by the fact that many spectral reference libraries can be poor at annotating natural products, as they often exhibit a bias toward industrial chemicals, primary metabolites, and other commercially available compounds.^88^ Hence, NMR methods, which require larger amounts of pure sample, remain indispensable.^89,90^ Advances in NMR analysis using ML have greatly decreased the amount of time needed to derive structural predictions.^91^

General recent advances in the field of metabolomics research have been made through concerted efforts on integrating data from different technologies and are now becoming available for NP research. MZMine3 is a tool for analyzing hybrid MS datasets that can integrate the analysis of raw spectral data from several different MS platforms.^92^ This fills the gap of large-scale MS^2^-based annotations, including applications for MS imaging analysis. This is highly relevant for NPs, which are often produced in spatially segregated regions of bacterial colonies, as illustrated by a recent study using MALDI-MS imaging in *in vitro* experiments using a bespoke membrane on an agar plate to obtain clear spatial data while reducing the background of smaller molecules.^93^

Collaboration in the NP field has yielded many key community tools for MS analysis, such as the Global Natural Products System (GNPS).^94^ GNPS generates molecular networks for user-submitted MS^2^ data that can be compared to >490,000 user-submitted MS^2^ spectra in its MassIVE database.^95,96^ There are approximately 50 software tools incorporated into the GNPS ecosystem, *e.g.*, microbeMASST, which can connect MS^2^ data to specific organisms.^88^ The use of FastSearch, originally a tool for proteomics, enables microbeMASST to search the GNPS/MassIVE repository at a rate orders of magnitude higher than MASST.^97^ Its outputs include taxonomic trees which connect the spectral data to the specific organisms in which matching spectral data have previously been detected, though the resolution for the taxonomy is not always at the strain level. MS2LDA has recently made its Mass2Motifs workflow, a tool which outputs biochemically relevant substructures, compatible with the GNPS ecosystem.^98,99^ A thorough review of these and other spectral analysis approaches can be found in the work of Zdouc and colleagues.^100^

## Learn

A key aspect of ML being used for NP research lies in the ability to learn from the many databases of known chemical structures of NPs and other chemicals. These datasets have been used as inputs for graph neural network (GNN) models which read in the chemical structures as graphs with atoms as nodes and atomic bonds as the edges connecting nodes. When combined with the results of high-throughput antibiotic activity screens, this approach has led to the discovery of antibiotic activity for several compounds that had not previously been known to have antibiotic activity, though not all of these have been NPs. Halicin^6^ and abaucin^101^ are two highly publicized drugs which had previously been characterized in the Drug Repurposing Hub^102^ and ZINC15^103^ libraries yet were only recently found to display antimicrobial properties after analysis using GNN models. Similar strategies should be applicable to NP research, and indeed, several related efforts have already discovered NPs which have displayed antibiotic activity against antibiotic-resistant strains.^104,105^ The vast amount of structural data for known NP molecules can also be leveraged to develop synthetic NP-like molecules, but additional work will be required to design bespoke (bio)chemicals and create bioactive neo-NPs on demand.^106^

The increasingly abundant omics data reported in the literature can be used to generate improved genome-scale metabolic models (GEMs) of chassis organisms.^107^ There are now GEMs available for many industrially relevant microorganisms tailored to specific use cases, such as production of a target NP or the use of a specific feedstock, as detailed in a thorough review by Han and colleagues.^108^ The use of GEMs for NP engineering can involve testing knockouts of specific genes *in silico* in order to maximize target NP production *in vitro*, as was done to optimize oleanolic acid production in *S. cerevisiae*.^109^ These approaches are highly beneficial when attempting to rationally engineer nonmodel organisms, as shown by recent work in the development of a GEM for *Vibrio natriegens*, which is an attractive industrial bacterium due to its high growth rate.^110^

## Responsible Research and Innovation

As shown in the preceding sections, NP research can rely heavily on the concepts and methods of synthetic biology to produce and characterize its products. Synthetic biology itself has promised to enable a greener and more sustainable alternative to several industrially relevant chemical processes.^111−113^ Fulfilling this promise will require careful techno-economic analysis (TEA) and full life-cycle assessment (LCA),^114,115^ which are not yet available for most NPs but will be a critical step when upscaling NP production to an industrial level.^116^

Early examples of TEA and LCA in the NP domain include a recent application to different photobioreactors to determine which non-open-air bioreactor could work best for microalgae producing ketocarotenoids.^117^ Another recent paper focused on comparing different corn-based feedstocks for shikimic acid bioproduction and concluded that a key factor was the economics of alternate uses of each feedstock.^114^ Work by Zhao and colleagues on different synthesis pathways of vanillin production, which identified electricity usage as the major determinant of environmental burden, highlights the need for more data to best estimate large-scale production parameters as well as the substantial context dependence of any such analysis, *e.g.*, as a result in dramatic differences of the modes of electricity generation.^118^

An impediment to using LCA or TEA can be a lack of information to guide the decision-making process at an early stage.^119^ For some NP production pathways, product yield is still too small to utilize industrially relevant parameters in the production system, and NP production, especially for novel antimicrobials, is often not performed at an industrial level due to the shrinking number of major pharmaceutical companies investing in antibiotics development.^120^ Nevertheless, it is possible to develop production systems already at the laboratory proof-of-concept stage in such a way that future large-scale applications are taken into account, as shown in a recent study using electrodialysis in the purification of the high-value antimicrobial peptide nisin and evaluating its benefits in a circular-economy framework.^121^ To facilitate the early anticipation of potential impacts of synthetic biology and to guide the choice between alternative production methods, the recently published Early Rapid Sustainability Assessment (ERSA) seeks to minimize the *a priori* information needed to perform such predictions.^122^ It introduces a framework that enables early-stage research to conduct a risk analysis for future scale-ups of an experimental workflow. This process has been tested across a range of NP fermentation processes and presents an early-stage tool for approximating the environmental impacts of experiments. ERSA thus provides an important tool for conscientiously developing NP fermentation processes while they are still on the scale of academic research and before technology lock-ins have occurred.
